# PtIr–WO_3_ nanostructured alloy for electrocatalytic oxidation of ethylene glycol and ethanol

**DOI:** 10.1007/s10008-014-2493-0

**Published:** 2014-05-10

**Authors:** Magdalena Murawska, James A. Cox, Krzysztof Miecznikowski

**Affiliations:** 1 Department of Chemistry, University of Warsaw, Pasteura 1, PL-02-093 Warsaw, Poland; 2 Department of Chemistry and Biochemistry, Miami University, Oxford, OH 45056 USA

**Keywords:** PtIr nanoparticles, Tungsten oxide, Ethylene glycol electrooxidation, Ethanol oxidation, Electrocatalysis

## Abstract

In this article, we characterized tungsten oxide-decorated carbon-supported PtIr nanoparticles and tested it for the electrooxidation reactions of ethylene glycol and ethanol. Phase and morphological evaluation of the proposed electrocatalytic materials are investigated employing various characterization techniques including X-ray diffraction (XRD) and transmission electron microscopy (TEM). Electrochemical diagnostic measurements such as cyclic voltammetry, chronoamperometry, and linear sweep voltammetry revealed that the tungsten oxide-modified PtIr/Vulcan nanoparticles have higher catalytic activity for ethylene glycol and ethanol electrooxidation than that of PtIr/Vulcan. A significant enhancement for electrooxidation of CO-adsorbate monolayers occurred in the presence of a transition metal oxide relative to that of pure PtIr/Vulcan electrocatalyst. The likely reasons for this are modification on the Pt center electronic structure and/or increasing the population of reactive oxo groups at the PtIr/Vulcan electrocatalytic interface in different potential regions.

## Introduction

An important task in the twenty-first century is to further develop fuel cells as alternative electrochemical devices for efficient generation of electricity. The low-temperature acid-type systems, such as hydrogen-oxygen polymer electrolyte membrane fuel cells and direct alcohol fuel cells, are, at present, the most commonly studied devices in many laboratories worldwide [1, 2]. Among organic compound fuels for anodic reactions in fuel cells, methanol has been historically most extensively studied [3], and more recently, other short chain liquid fuels such as ethanol and ethylene glycol have become important [4–11]. In the case of direct methanol fuel cells (DMFC), slow electrode kinetics of methanol oxidation and methanol adsorption products (which is mainly CO_ads_) poisoning the surface of Pt electrode at low temperature still hamper application [3, 12–15]. As a result, new fuels and new catalysts remain important research topics.

Polyhydric and monohydric (except methanol) alcohols, such as ethylene glycol and ethanol, have been proposed as potential FC fuels which are much less volatile and less toxic than methanol. Moreover, both alcohols have some of the largest volumetric energy densities, and they involve the transfer of a number of electrons that set a practical challenge for the effectiveness of catalysts. They also show lower permeability through membranes (lower crossover effect) [16–18]. However, the electrochemical oxidation of short chain alcohol (monohydric or polyhydric alcohols) is much more complex than, for example, H_2_ oxidation. The main challenge generally for electrooxidation of polyhydric and monohydric alcohols to carbon dioxide is associated with the cleavage of the C–C bond for complete conversion. Due to incomplete oxidation, various intermediate species from electrooxidation in both alcohols are observed.

It is well established that Pt is rated as the most active material for oxidation of small organic molecules in acidic media, but poisoning by the intermediate by-product of CO-adsorbed species of the binary or ternary platinum-based alloys with Ru, Sn, Rh, Pb, W, or Mo [19–27] was proposed to enhance the electrooxidation activity toward alcohols. Alternatively, Ir has also been employed as a co-metal for platinum-based catalysts in unitized polymer electrolyte fuel cells because of its high stability and resistance to corrosion [28, 29]. The presence of Ir, particularly IrO_2_, enhances the electrooxidation of methanol in direct methanol fuel cells due to providing a large number of OH groups that are adsorbed at relatively low potentials [30–34]. Moreover, Cao et al. reported that the addition of Ir into Sn showed to be a promising alternative for Pt-based catalysts for ethanol electrooxidation [35]. Tremiliosi-Filho et al. demonstrated a positive effect for ethanol oxidation in which IrO_2_ was incorporated to platinum-based catalysts [36]. Recently, Adzic et al. revealed that the presence of a high content of Ir atoms into ternary catalyst PtIr/SnO_2_/C enhances the complete electrooxidation of ethanol to CO_2_ at a relatively low-onset potential [34]. Furthermore, PtIr catalysts have been utilized for electrooxidation of ethylene glycol with positive results [37, 38].

One of the numerous approaches to increase the electrocatalytic activity of platinum-based catalysts toward the oxidation of small organic compounds is the use of transition metal oxides as support systems for catalytic metal sites. The presence of transition metal oxides in the neighborhood of catalytic sites of noble metal catalysts results in an increasing population of –OH groups at low potentials, thereby mitigating CO poisoning of catalytically active platinum centers, possibly facilitating the cleavage of C–H bonds as well as in a weakening of C–C bonds. This assumption is in accord with reports in which a significant improvement in oxidation of small organic molecules with metal oxides (e.g., WO_3_, MoO_3_, TiO_2_, ZrO_2_, V_2_O_5_, and CeO_2_) modified by Pt-based alloy catalysts has been observed [8, 9, 39–43].

The present work will concentrate on the preliminary investigation of a carbon-supported PtIr-based anodic catalyst with tungsten oxide as the additive prepared by the adsorption of tungsten acid. Not only the peak current value during cyclic voltammetry (CV) tests in a broad potential region, but also the more specific performance in the low potential region will be evaluated from a more comprehensive point of view. Thus, the electrocatalytic activity toward ethanol and ethylene glycol oxidation in comparison with that of PtIr/C and WO_3_-modified PtIr/C catalysts will be evaluated by CV, linear sweep voltammetry (LSV), and chronoamperometry (CA) methods.

## Experimental

All chemicals were commercial materials of analytical grade purity that were obtained from Premetek PtIr/C nanoparticles (20 % on Vulcan XC-72, Pt:Ir 1:1). Solutions were prepared using doubly distilled and subsequently deionized (Millipore Milli-Q) water. Argon was used to de-aerate the solutions and to keep an oxygen-free atmosphere over the solution during the measurements. Some characteristics of catalytic particles were obtained using a LIBRA 120 transmission electron microscope (TEM) operating at 120 kV. Samples for TEM measurements were prepared by depositing drops of colloidal solutions of nanoparticles onto 400-mesh copper grids supporting a Fromvar film (Agar Scientific) and, later, drying them in ambient laboratory conditions (temperature, 20 ± 2 °C) for 24 h prior to TEM analysis. X-ray diffraction (XRD) patterns of the catalysts were obtained with a Bruker D8 Discover operating with a Cu X-ray tube (1,5406 Å) and Vantec (linear) detector (*k* = 1.5406 Å).

Electrochemical characterization was performed in a three-electrode, single compartment cell. The working electrode was glassy carbon, and the counter electrode was carbon rod. As a rule, all potentials in the present work were measured versus a K_2_SO_4_-saturated Hg_2_SO_4_ reference electrode and were recalculated and reported versus the reversible hydrogen electrode (RHE). CH Instruments 750 A workstations were used for all electrochemical measurements.

The catalyst layer was fabricated through modification of the working electrode by immobilization of PtIr/C nanoparticles. WO_3_ modification of the PtIr/C catalyst was in accordance with the procedure described in our previous papers [8, 9]. Briefly, a solution of tungstic acid was prepared by passing an aqueous solution of 0.05 mol dm^−3^ Na_2_WO_4_ through a proton exchange resin. In a typical procedure, selected amount of PtIr/C catalyst was added to 2 cm^3^ of 0.05 mol dm^−3^ aqueous solution of tungstic acid. The resulting suspension was stirred for 24 h. During that process, the PtIr/C nanoparticles interacted with tungstic acid to form tungsten oxide or hydrogen tungsten oxide bronzes. The supernatant solution was centrifuged and replaced with water in order to obtain a colloidal solution of tungsten oxide-modified PtIr/C nanoparticles that was stable for months. The Pt-to-tungsten ratio in the given catalyst system was determined with X-ray fluorescence (XRF); the targeted ratio was approximately 1:1.

To prepare a homogeneous catalyst layer on the glassy carbon working electrode surface, a 5-μl aliquot of the catalyst dispersion was deposited using a micropipette (the nominal loading of catalyst was approximately 160 μg cm^−2^) and allowed to dry under ambient conditions. Prior to this step, the suspensions were treated in an ultrasonic bath for 5 min. When the catalyst layers had dried, 2 μl of Nafion (0.02 % alcoholic solution) was dropped on top of the glassy carbon electrode surface covered with the catalyst and dried at room temperature. Prior to the electrooxidation processes, the catalytic electrodes were scanned with 25 complete oxidation/reduction cycles between 0.0 and 0.8 V in 0.5 mol dm^−3^ H_2_SO_4_ at 50 mV s^−1^ scan rate.

The CO-stripping experiments were carried out in 0.5 mol dm^−3^ H_2_SO_4_ electrolyte utilizing the glassy carbon electrode substrate onto which surface the appropriate catalyst was introduced. As a rule, a few cyclic voltammetric measurements (at 50 mV s^−1^) were recorded in the potential range from 0.0 to 0.8 V in the deoxygenated electrolyte. The CO-saturated solution was prepared by flowing pure CO (from Air Liquide) through the electrolyte for 10 min. The CO adsorption process that was employed (mainly on the surface of catalytic Pt nanocenters) was achieved by underpotential control at 0.1 V versus RHE for 5 min, after which the dissolved CO was removed from the electrolyte by bubbling argon for 30 min maintaining the applied potential (0.1 V), in order to have a solution free of CO. Then, the adsorbed CO monolayer was stripped by recording three cyclic voltammetric scans in the potential range from 0.0 to 0.9 V at a scan rate of 10 mV s^−1^. The measurements using these catalysts were repeated three or four times with freshly prepared electrodes, and the average results are presented here.

## Results and discussion

**Fig. 1:**
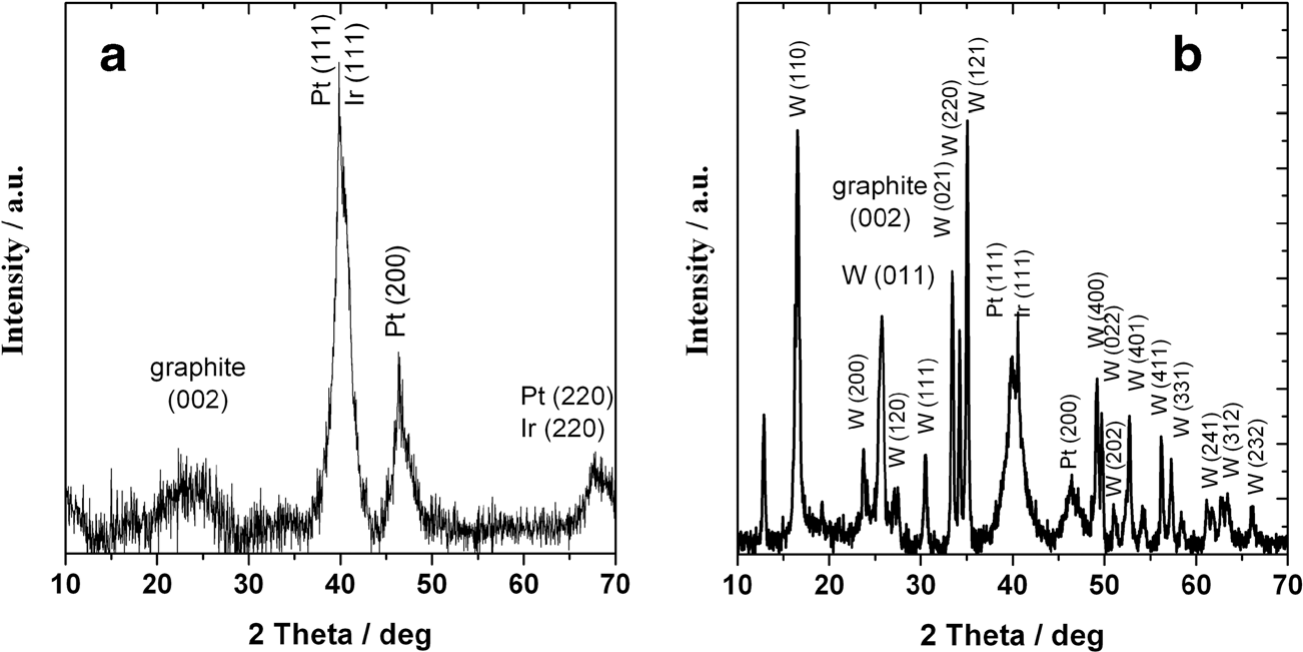
XRD diffractograms of PtIr/Vulcan (**a**) and WO_3_-modified PtIr/Vulcan (**b**)

The X-ray patterns of the PtIr/Vulcan nanoparticles in the presence and absence of the WO_3_ modifier are shown in Fig. 1a, b. The broad diffraction peak centered at 20.0–25.0° in all the XRD pattern is attributed to the hexagonal carbon support [44]. In the case of unmodified PtIr/Vulcan nanoparticles, the other characteristic peaks at ca. 39.5°, 46.3°, and 67.7° correspond to the Pt lattice planes (PCPDF 04-0802) [45, 46]. The diffractogram of the WO_3_-modified PtIr/Vulcan electrocatalyst shows peaks at 2*θ* = 40.5°, 46.9°, and 66.2°, which are associated with Pt reflections and the signals that originated from WO_3_ (PCPDF 43-0679). The results indicate that Pt fcc is the main crystalline phase in the catalysts and that the presence of tungsten species resulted in the formation of crystalline aggregates. A peak shift is observed which could indicate the interaction between PtIr/Vulcan alloy and tungsten oxide. Of the XRD patterns, none were metallic Ir, or iridium oxide diffraction peaks have been observed because Pt and Ir metals have similar diffraction peak positions and crystalline structures [33, 36]. To estimate the average particle sizes from Scherrer’s equation, the Pt peak (at 2*θ* = 39.5°) was used [46]. The latter peak was chosen because it is located in a region where there are no interferences from the carbon support. The average Pt particle sizes were obtained from the position and the full-width at half-maximum values of the Pt peak (at 2*θ* = 39.5°). The values were in the ranges from 4 to 6 and 6 to 8 nm for unmodified and modified PtIr/Vulcan nanoparticles, respectively. Higher values of the average particle sizes for the WO_3_-modified PtIr/Vulcan can be interpreted in terms of deposition of the WO_3_ crystalline monoclinic structure.

**Fig. 2:**
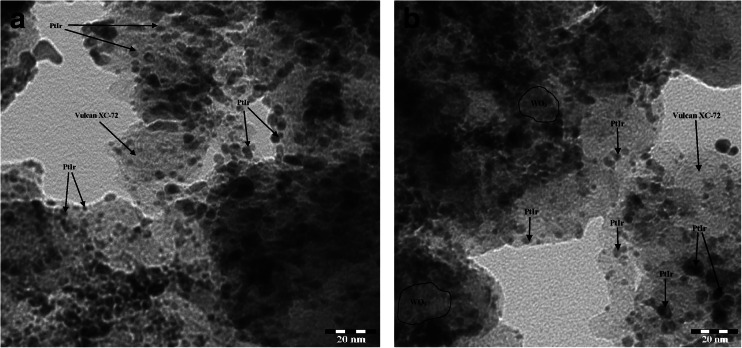
Low magnification micrographs (TEM) of PtIr/Vulcan (**a**) and WO_3_-modified PtIr/Vulcan (**b**)

In order to get more information about the size, morphology, and distribution of nanoparticles on the carbon material, TEM analysis was performed. Figure 2 shows the TEM images and distributions of the series of PtIr/C and WO_3_-modified PtIr/C nanoparticles. Low magnification images show that the supported material was predominantly irregular spheres or spheroids of bimetallic nanoparticles that were homogeneously dispersed on the carbon (Vulcan XC-72) surfaces. The average particle sizes lie in the narrow range of 5–8 nm with a standard deviation of 1 nm, which is in agreement with the XRD results for WO_3_-modified PtIr/Vulcan and unmodified PtIr/Vulcan catalysts.

**Fig. 3:**
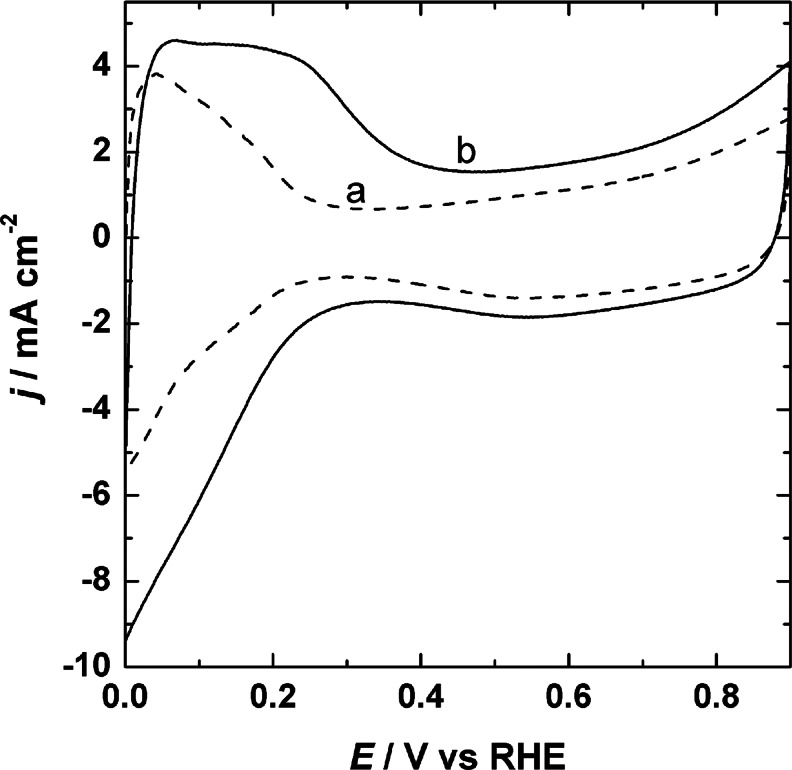
Cyclic voltammetric responses of PtIr/Vulcan (*a*) and WO_3_-modified PtIr/Vulcan (*b*) catalytic systems in 0.5 mol dm^−3^ H_2_SO_4_. Scan rate = 10 mV s^−1^

For initial electrochemical characterization, cyclic voltammetric curves of the PtIr/Vulcan and the WO_3_-modified PtIr/Vulcan nanoparticles deposited on glassy carbon electrode were obtained in 0.5 mol dm^−3^ sulfuric acid-supporting electrolyte (Fig. 3). In both cases, slight changes in the shape or current values of the cyclic voltammetric curves are observed. In the hydrogen adsorption/desorption region (between 0.0 and 0.4 V versus RHE) for the all proposed catalysts, some changes in the voltammetry are seen because of the dependence on the surface composition. The voltammogram for unmodified PtIr/Vulcan (Fig. 3a) is characterized by a single large peak in the hydrogen adsorption/desorption region, whereas the electrochemical behavior of electrodes made from the WO_3_-modified PtIr/Vulcan nanoparticles (Fig. 3b) shows two peaks in this region. Moreover, in the double layer region, which is between 0.4 and 0.7 V versus RHE, significant currents are recorded. This behavior is typical of electrocatalysts composed of transition metals dispersed on a carbon black support [36]. It is apparent from Fig. 3b that the oxidation peak appearing at about 0.2 V most likely reflects the intercalation of protons in tungsten oxide (WO_3_) and tends to overlap with the hydrogen adsorption/desorption region of bare platinum (at a potential lower than 0.4 V). This interpretation is difficult to prove because of the problem of unambiguously distinguishing contributions from the reversible reduction of tungsten oxide to hydrogen tungsten bronzes from the abovementioned hydrogen adsorption and desorption peaks originating from Pt and Ir.

**Fig. 4:**
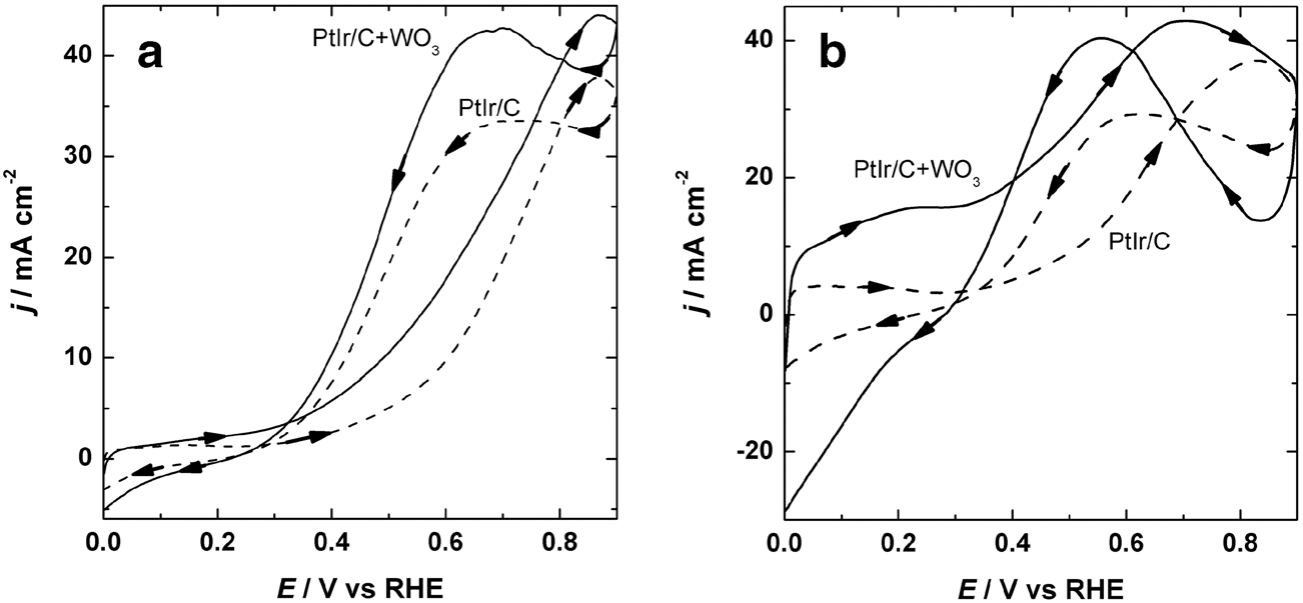
Cyclic voltammetric responses for oxidation of 0.5 mol dm^−3^ ethanol (**a**) with PtIr/Vulcan (*a*) and WO_3_-modified PtIr/Vulcan (*b*) and 0.5 mol dm^−3^ ethylene glycol (**b**) with PtIr/Vulcan (*a*) and WO_3_-modified PtIr/Vulcan (*b*) catalysts. Electrolyte = 0.5 mol dm^−3^ H_2_SO_4_. Scan rate = 10 mV s^−1^

The behavior of the proposed catalytic layers was tested toward ethanol and ethylene glycol electrooxidation processes. Representative cyclic voltammograms of these species at both PtIr/Vulcan and WO_3_-modified PtIr/Vulcan electrocatalysts deposited on glassy carbon electrode are shown in Fig. 4. The cyclic voltammograms of ethanol (Fig. 4a) and ethylene glycol (Fig. 4b) obtained at the PtIr/Vulcan and WO_3_-modified PtIr/Vulcan surface, respectively, show well-defined peaks for both forward and reverse scans in the investigated potential region. In the forward scan, the ethanol oxidation current at PtIr/Vulcan catalysts starts (at 0.3 V) and reaches the peak at 0.87 V, which is located at the same potential as compared to the WO_3_-modified PtIr/Vulcan, as illustrated in Fig. 4a. In the reverse scan, a single peak is developed at 0.7 V, which can be attributed to the oxidative decomposition of by-products [16].

Similar measurements to those presented in Fig. 4b were performed with ethylene glycol. The cyclic voltammograms of WO_3_-modified PtIr/Vulcan and bare PtIr/Vulcan in the presence of ethylene glycol exhibited no significant oxidation current up to 0.27 and 0.35 V, respectively. Two distinct peaks were observed in the potential range between 0.4 and 0.9 V in comparison to previous literature reports [36–38], and these processes can be attributed primarily to the oxidation of adsorbed organic species. In the case of electrooxidation of ethylene glycol, various intermediate oxidation products can be expected according to spectroscopic data [47–52] including CO, glycol aldehyde, glycolate, glyoxylate, oxalate, and formate. This is in contrast to the data obtained with ethanol, the oxidation of which produces only CH_2_COOH, CO_2_, and CH_3_CHO [53].

Generally, in the presence of both fuels, the current density in the hydrogen region decreases in comparison to the voltammograms obtained in the supporting electrolyte alone due to their adsorption. Additionally, Fig. 4 displays that in both cases, the current density values are higher in the tested potential range confirming the enhancement of the electrooxidation of ethanol and ethylene glycol by the presence of the metal oxide WO_3_. In addition, the onset potentials of ethanol and ethylene glycol oxidation shift toward more negative values which is especially pronounced in the case of ethylene glycol oxidation. This can be explained by the fact that transition metal oxides (e.g., WO_3_ and related compounds) are known to activate interfacial water molecules (from –OH groups on WO_3_) at lower potentials which, in turn, promote the removal of poisoning species from the noble metal catalyst [8, 9, 14, 54–60].

**Fig. 5:**
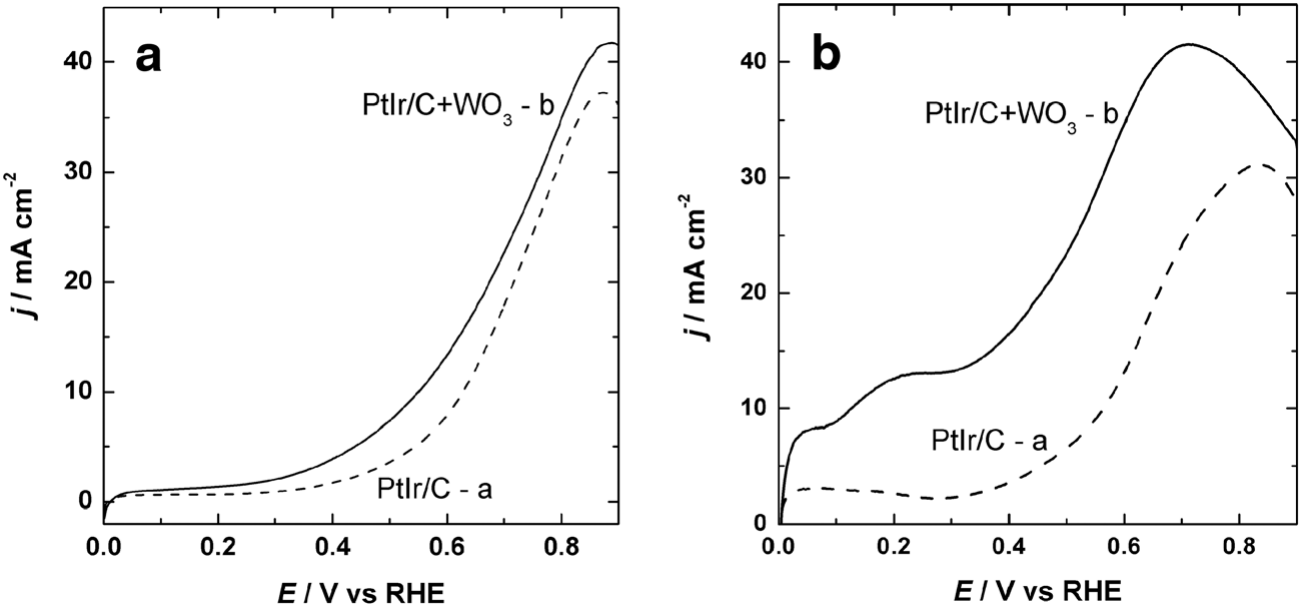
Linear scan voltammetry responses for oxidation of 0.5 mol dm^−3^ ethanol (**a**) with PtIr/Vulcan (*a*) and WO_3_-modified PtIr/Vulcan (*b*) and 0.5 mol dm^−3^ ethylene glycol (**b**) with PtIr/Vulcan (*a*) and WO_3_-modified PtIr/Vulcan (*b*) catalyst at 10 mV s^−1^ scan rate. Electrolyte = 0.5 mol dm^−3^ H_2_SO_4_

Figure 5 exhibits background-subtracted linear scan voltammograms (LSVs) for ethanol and ethylene glycol electrooxidation on PtIr/Vulcan and WO_3_-modified PtIr/Vulcan electrocatalysts deposited on glassy carbon substrate recorded in the potential range of 0.0–0.9 V. As can be seen for both fuels, the shape of the LSV curves is almost the same in the examined potential range. The only difference is that the current densities are higher for WO_3_-modified PtIr/Vulcan nanoparticles than those recorded on bare PtIr/Vulcan nanoparticles. This behavior is obvious in the case of ethylene glycol oxidation. The LSV experiments also confirm the shifting of the onset potential for ethanol and ethylene glycol electrooxidation on WO_3_-modified PtIr/Vulcan catalysts toward lower potential values. It is likely that the increases of the oxidation current densities observed in the LSV curves are associated with the addition of tungsten oxide. The decrease of the onset potential of ethylene glycol and ethanol oxidation is due to activation of interfacial water molecules forming –OH species at lower anodic potential than the bare catalyst.

The effect of temperature for ethanol and ethylene glycol electrooxidation in bare and WO_3_-modified PtIr/Vulcan nanoparticles was also investigated. When the temperatures increase from 10 to 50 °C, the oxidation current densities for both become higher. Based on the Arrhenius equation, the activation energy (45 and 49 kJ mol^−1^, respectively) can be determined from the slope calculated by linear regression by plotting ln(*j*) as a function of reciprocal of temperature (figure not shown). The value of activation energy is in good agreement with a previous report on alcohol electrooxidation [61]. This observation suggests that the increase of temperature causes more facile oxidation kinetics and decreases poisoning by the intermediate species.

**Fig. 6:**
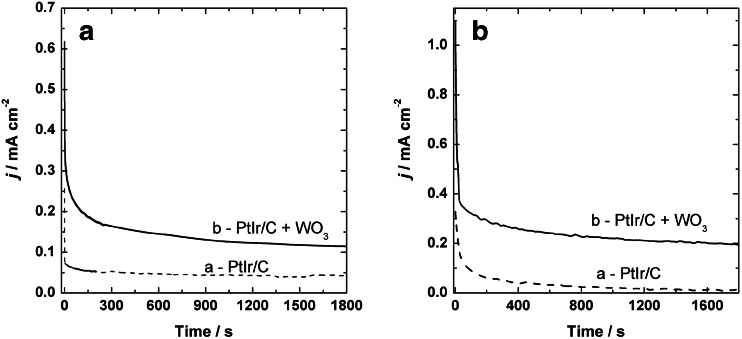
Chronoamperometric current-time responses (recorded at 0.3 V) for oxidation of 0.5 mol dm^−3^ ethanol (**a**) with bare PtIr/Vulcan (*a*) and WO_3_-modified PtIr/Vulcan (*b*) and 0.5 mol dm^−3^ ethylene glycol (**b**) with PtIr/Vulcan (*a*) and WO_3_-modified PtIr/Vulcan (*b*) catalysts. Electrolyte = 0.5 mol dm^−3^ H_2_SO_4_

In order to evaluate the electrocatalytic activity and also the long-term stability of tungsten oxide-modified PtIr/Vulcan nanoparticles for ethanol and ethylene glycol oxidation, chronoamperometric measurements were performed at low potentials (Fig. 6). The polarization current density for the electrooxidation of both fuels on the investigated catalytic systems displays a rapid decrease in the first period of the experiment before reaching a stable value. The catalytic current developed for tungsten oxide-modified PtIr/Vulcan electrocatalysts for both ethanol and ethylene glycol always was significantly higher than those produced for bare PtIr/Vulcan nanoparticles in the tested time period. These results are consistent with those obtained by cyclic voltammetry. In the initial phase of the chronoamperometric experiments, it is likely that a higher number of free active sites are available for adsorbed ethanol or ethylene glycol molecules (fast kinetic rate reaction), and during the next few minutes (rate determining step), the amount of free catalyst sites is limited by poisoning by intermediate species, such as CO, CH_*x*_, CH_3_CHO, CH_3_COOH (for ethanol oxidation), glycol aldehyde, glycolate, glyoxylate, oxalate, and glycolate (for ethylene glycol). In this regard, the improvement of catalytic properties observed by introduction of WO_3_ on Pt-based nanoparticles surface can be associated with the oxophilic nature of tungsten oxide providing hydroxyl groups (–OH) on the oxide surface at lower potential, which promotes electrooxidation of the surface CO-poisoning intermediates species [8, 9, 54–60].

Regarding the stability of the electrocatalytic responses in the presence of ethylene glycol and ethanol, the long-term chronoamperometric and repetitive voltammetric measurements of WO_3_-modified PtIr/C and bare PtIr/C systems have been performed (not shown here) under the same conditions. In the potential range between 0.0 and 0.9 V, the catalytic peak currents decreased, remaining at 90 % than those of the first cycle after 100 cycles when the WO_3_-modified system has been used. During long-term chronoamperometric experiments (1 h), there was only 15 % decrease of catalytic currents in the case of tungsten oxides. In both experiments, no significant deactivation effect was observed that may imply dissolution of WO_3_.

**Fig. 7:**
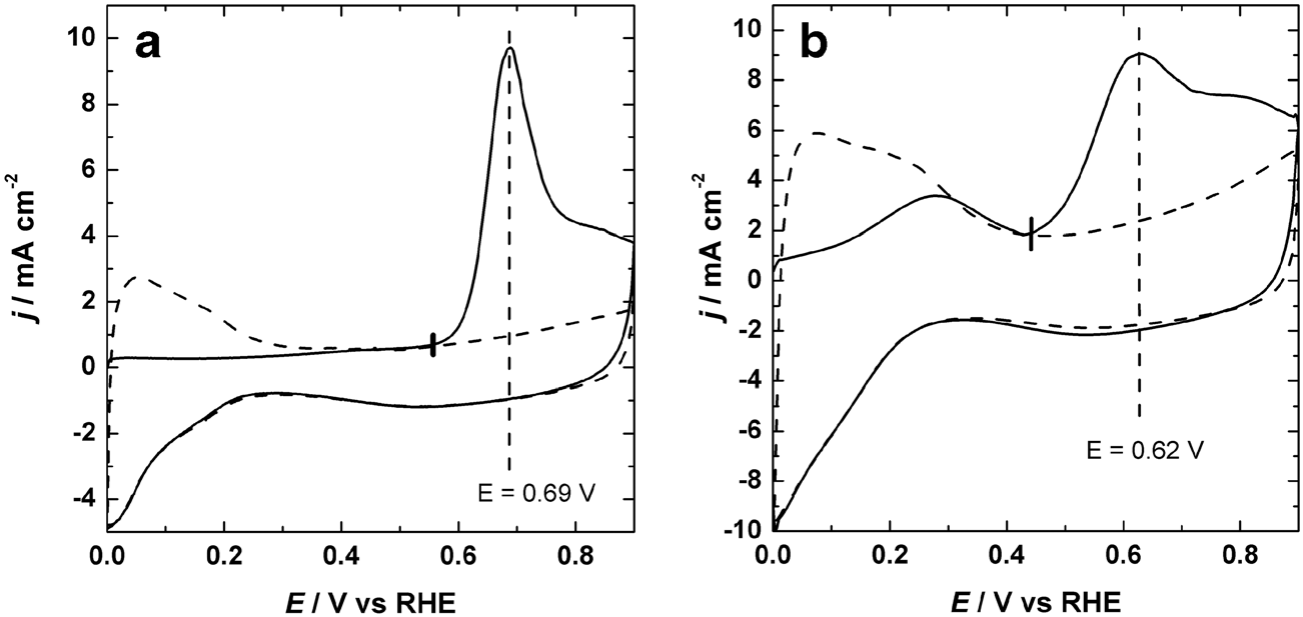
CO-stripping voltammograms recorded at 10 mV s^−1^ in 0.5 mol dm^−3^ H_2_SO_4_ for the PtIr/Vulcan (**a**) and WO_3_-modified PtIr/Vulcan (**b**) catalysts. CO adsorption was done at 0.1 V. *Solid curve* shows the first cycles, while the *dot curve* shows the second cycles

In order to gather information of the ability of CO_ads_ poisoning species to undergo oxidative desorption from the surface of the prepared Pt-based catalysts, CO-stripping voltammetry was performed. A typical CO_ads_-stripping curve on bare PtIr/Vulcan catalyst is presented in Fig. 7a. It is characterized by a single sharp and prominent CO_ads_ oxidation peak centered at 0.69 V with a CO_ads_ oxidation onset potential of 0.590 V. In contrast, for the WO_3_-modified PtIr/Vulcan catalysts, two separated CO_ads_ oxidation peaks with the position of the main peak potential at 0.62 V were found in the stripping voltammetric method (Fig. 7b). The onset potential for the main CO_ads_ oxidation peak starts near 0.49 V. It becomes broadened and shifts to a more negative potential (ca. 100 mV) versus the main CO_ads_ oxidation peak for the bare PtIr/Vulcan. For both catalysts, the hydrogen region was completely blocked by the full coverage with CO_ads_; the main CO-stripping (oxidation) peak appeared only during the first anodic cyclic, which indicates that all adsorbed CO was oxidized and removed from the surface under this condition. This observation is in good agreement with the previous reports for metal oxides (e.g., WO_3_, MoO_3_) [8, 9, 62, 63].

The presence of two peaks on the CO-stripping voltammetry has been described in the literature [64–68]. Two signals observed for oxidation of CO-adsorbed curve on the WO_3_-modified PtIr/Vulcan catalyst suggest that two active sites for CO_ads_ oxidation may exist, which are the likely case due to a variation in the interaction with the metal oxide. In a previous report, this effect for Pt-based electrode has been explained by the presence of at least two different types of CO adsorption products (linearly or bridged bound) which are characterized by different binding energies [69]. Moreover, the peak that appears at the lower potential may be ascribed the CO_ads_ oxidation on the surface of WO_3_-modified PtIr/Vulcan catalyst corresponding to the interaction between the Pt nanoparticles and tungsten oxide, whereas the peak at the higher potential may be attributed to adsorption on Pt nanoparticles at a large distance from tungsten oxide. Additionally, it cannot be excluded that this peak originates from the redox reaction of a tungsten species.

To estimate the electrochemically active surface area (*S*_A_), the CO-stripping voltammetry method was used. The hydrogen adsorption/desorption system cannot be used because of the overlap with the redox process of tungsten oxide. The CO-stripping voltammetry method relies on forming a monolayer of strongly adsorbed CO on the bare and modified PtIr/Vulcan catalysts. Generally, CO may form a linear, bridge bond to the surface leading to different numbers of electrons per site (2 and 1, respectively). Those two CO adsorption configurations are strongly influenced by the applied potential. A linear adsorption may dominate if the CO adsorption occurs at a potential close to 0 V. By analogy to these conditions, it was assumed that one monolayer of CO adsorbed on Pt (linked linearly) and that the coulombic charge required to oxidize adsorbed CO to CO_2_ is equal to 420 μC cm^−2^ [63, 70]. The charge value required to estimate the electrochemically active surface area was determined by integrating the main CO-stripping peaks. The obtained *S*_A_ values for the catalysts were 57 and 54 m^2^ g^−1^ for bare PtIr/Vulcan and WO_3_-modified PtIr/ Vulcan, respectively. It is reasonable to conclude that the electrochemical active surface area is not the major factor causing the difference of their catalytic activities for ethylene glycol and for ethanol electrooxidations. In other words, tungsten oxide species occupied slightly the electrochemically active surface area of modified PtIr/Vulcan nanoparticles. The same tendency has been noticed by others with different catalysts (e.g., Pt, PtSn, and PtRh) and with various transition metal oxides [57, 62, 63, 71]. These effects may be explained by the ability of tungsten oxide in contact with aqueous solutions to increase the population of surface hydroxyl groups which likely play a major role in the CO_ads_ removal [55]. Further work is needed to determine whether such catalysts are stable under test conditions for fuel cells to elucidate full reaction pathways.

## Conclusions

Herein, we demonstrate the enhancement of the activity of catalysts composed of bimetallic PtIr nanoparticles and tungsten oxide toward electrooxidation of ethylene glycol and ethanol. From both XRD and TEM results, the average particle sizes were found to be in the range of 4–8 nm and uniformly dispersed on glassy carbon. The adsorbed layer of tungsten oxide on PtIr/Vulcan nanoparticles increases the catalytic currents and decreases the onset potentials for electrooxidation of ethylene glycol and ethanol. The above electrochemical measurements confirmed that the presence of tungsten oxide on the surface is beneficial in the electrooxidation of ethylene glycol and ethanol. The results also showed that the activity of the PtIr/Vulcan nanoparticles for oxidation of poisoning species (CO_ads_) is higher in the presence of tungsten oxide. The activation effect may involve direct specific interactions (chemical or electronic) between WO_3_ and both Pt and Ir metals.

## References

[1] Antolini E (2009). Appl Catal B Environ.

[2] Zhang J (2008). PEM fuel cell electrocatalysts and catalyst layers fundamentals and applications.

[3] Liu H, Zhang J (2009) Electrocatalysis of direct methanol fuel cells. Wiley-Vch

[4] Demarconnay L, Brimaud S, Coutanceau C, Leger JM (2007). J Electroanal Chem.

[5] Serov A, Kwak C (2010). Appl Catal B Environ.

[6] Halseid MC, Jusys Z, Behm RJ (2010). J Electroanal Chem.

[7] Feng YY, Yin WP, Li Z, Huang CD, Wang YX (2010). Electrochim Acta.

[8] Miecznikowski K, Kulesza PJ (2011). J Power Sources.

[9] Miecznikowski K (2012). J Solid State Electrochem.

[10] Kowal A, Li M, Shao M, Sasaki K, Vukmirovic MB, Zhang J, Marinkovic NS, Liu P, Frenkel AI, Adzie RR (2009). Nature Mater.

[11] Li ZY, Liang YJ, Jiang SP, Shan XD, Lin ML, Xu CW (2012). Fuel Cells.

[12] Kulesza PJ, Grzybowska B, Malik MA, Chojak M, Miecznikowski K (2001). J Electroanal Chem.

[13] Vijayaraghavan G, Gao L, Korzeniewski C (2003). Langmuir.

[14] Barczuk PJ, Tsuchiya H, Macak JM, Schmuki P, Szymanska D, Makowski O, Miecznikowski K, Kulesza PJ (2006). Electrochem Solid-State Lett.

[15] Cheng Y, Jiang SP (2013). Electrochim Acta.

[16] Livshits V, Peled E (2006). J Power Sources.

[17] Liu H, Qiqan W, Wilkinson DP, Shen J, Wang H, Zhang J (2006). J Power Sources.

[18] Jablonski A, Lewera A (2012). Appl Catal B-Environ.

[19] Lamy C, Lima A, LeRhun V, Delime F, Countanceau C, Leger JM (2002). J Power Sources.

[20] Jusys Z, Schmidt TJ, Dubau L, Lasch K, Jorissen L, Garche L, Behm RJ (2002). J Power Sources.

[21] Neto AO, Giz MJ, Perez J, Ticianelli EA, Gonzalez ER (2002). J Electrochem Soc.

[22] Mann J, Yao N, Bocarsly AB (2006). Langmuir.

[23] Wang H, Jusys Z, Behm RJ (2006). J Power Sources.

[24] Colmati F, Antolini E, Gonzalez ER (2006). J Power Sources.

[25] Sen Gupta S, Datta J (2006). J Electroanal Chem.

[26] Li GC, Pickup PG (2006). Electrochim Acta.

[27] Neto AO, Dias RR, Tusi MM, Linardi M, Spinace EV (2007). J Power Sources.

[28] Ioroi T, Yasuda K (2005). J Electrochem Soc.

[29] Fatih K, Neburchilov V, Alzate V, Neagu R, Wang H (2010). J Power Sources.

[30] Reetz MT, Lopez M, Grunert W, Vogel W, Mahlendorf F (2003). J Phys Chem B.

[31] Tsaprailis H, Birss VI (2004). Electrochem Solid State Lett.

[32] Chen A, La Russa DJ, Miller B (2004). Langmuir.

[33] Liao S, Holmes KA, Tsaprailis H, Birss VI (2006). J Am Chem Soc.

[34] Li M, Cullen DA, Sasaki K, Marinkovic NS, More K, Adzic RR (2013). J Am Chem Soc.

[35] Cao L, Sun G, Li H, Xin Q (2007). Electrochem Commun.

[36] Ribeiro J, dos Anjos DM, Kokoh KB, Coutanceau C, Leger JM, Olivi P, de Andrade AR, Tremiliosi-Filho G (2007). Electrochim Acta.

[37] Neto AO, Vasconcelos TRR, Da Silva RWRV, Linardi M, Spinac EV (2005). J Appl Electrochem.

[38] Raghuram C, Keith S (2007). J Appl Electrochem.

[39] Maiyalagan T, Khan FN (2009). Catal Commun.

[40] Song H, Qiu X, Li F (2009). Appl Catal A-General.

[41] Anjos DMD, Kokoh KB, Leger JM, Andrade ARD, Olivi P, Tremiliosi-Filho G (2006). J Appl Electrochem.

[42] Ou DR, Mori T, Togasaki H, Takahashi M, Ye F, Drennan J (2011). Langmuir.

[43] Hepel M, Kumarihamy I, Zhong CJ (2006). Electrochem Commun.

[44] Wang J, Yin G, Shao Y, Zhang S, Wang Z, Gao Y (2007). J Power Sources.

[45] Zhou W, Zhou Z, Song S, Li W, Sun G, Tsiakaras P, Xin Q (2003). Appl Catal B Environ.

[46] Radmilovic V, Gasteiger HA, Ross PN (1995). J Catal.

[47] Hahn F, Beden B, Kadirgan F, Lamy C (1987). J Electroanal Chem.

[48] Christensen PA, Hammet A (1989). J Electroanal Chem.

[49] Belgsir EM, Bouhier E, Yei HE, Kokoh KB, Beden B, Huser H, Leger JM (1991). Electrochim Acta.

[50] Dailey A, Shin J, Korzeniewski C (1998). Electrochim Acta.

[51] Fan YJ, Zhou ZY, Zhen CH, Fan CJ, Sun SG (2004). Electrochim Acta.

[52] Schnaidt J, Heinen M, Jusys Z, Behm RJ (2012). J Phys Chem C.

[53] Heinen M, Jusys Z, Behm RJ (2010). J Phys Chem C.

[54] Grgur BN, Markovic NM, Ross PN (1998). J Phys Chem B.

[55] Bock C, MacDougall B (2002). Electrochim Acta.

[56] Yang LX, Bock C, MacDougall B, Park J (2004). J Appl Electrochem.

[57] Manzo-Robledo A, Boucher AC, Pastor E, Alsono-Vante N (2002). Fuel Cells.

[58] Jayaraman S, Jaramillo TF, Baeck SH, McFarland EW (2005). J Phys Chem B.

[59] Maillard F, Peyrelade E, Soldo-Olivier Y, Chatenet M, Chaınet E, Faure R (2007). Electrochim Acta.

[60] Micoud F, Maillard F, Bonnefont A, Job N, Chatenet M (2010). Phys Chem Chem Phys.

[61] Chojak Halseid M, Jusys Z, Behm RJ (2010). J Electroanal Chem.

[62] Ma L, Zhaoa X, Si F, Liu C, Liao J, Liang L, Xing W (2010). Electrochim Acta.

[63] Wang ZB, Zuo PJ, Yin GP (2009). Fuel Cells.

[64] Wolter O, Heitbaum J (1984). Ber Bunsenges Phys Chem.

[65] Sobkowski J, Czerwinski A (1985). J Phys Chem.

[66] Leiva EPM, Santos E, Iwasita T (1986). J Electroanal Chem.

[67] Visscher W, Gootzen JFE, Cox AP, van Veen JAR (1998). Elecrochim Acta.

[68] Couto A, Rincon A, Perez MC, Guttierrez C (2001). Electrochim Acta.

[69] Siwek H, Lukaszewski M, Czerwinski A (2008). Phys Chem Chem Phys.

[70] Santiago EI, Camara GA, Ticianelli EA (2003). Electrochim Acta.

[71] Jiang L, Colmenares L, Jusys Z, Sun GQ, Behm RJ (2007). Electrochim Acta.

